# Metabolomics‐driven discovery of therapeutic targets for cancer cachexia

**DOI:** 10.1002/jcsm.13465

**Published:** 2024-04-21

**Authors:** Pengfei Cui, Xiaoyi Li, Caihua Huang, Donghai Lin

**Affiliations:** ^1^ College of Food and Pharmacy Xuchang University Xuchang China; ^2^ Xuchang Central Hospital Xuchang China; ^3^ Research and Communication Center of Exercise and Health Xiamen University of Technology Xiamen China; ^4^ Key Laboratory for Chemical Biology of Fujian Province, MOE Key Laboratory of Spectrochemical Analysis and Instrumentation, College of Chemistry and Chemical Engineering Xiamen University Xiamen China

**Keywords:** Cancer cachexia, Metabolic pathway, Metabolomics, Therapeutic target

## Abstract

Cancer cachexia (CC) is a devastating metabolic syndrome characterized by skeletal muscle wasting and body weight loss, posing a significant burden on the health and survival of cancer patients. Despite ongoing efforts, effective treatments for CC are still lacking. Metabolomics, an advanced omics technique, offers a comprehensive analysis of small‐molecule metabolites involved in cellular metabolism. In CC research, metabolomics has emerged as a valuable tool for identifying diagnostic biomarkers, unravelling molecular mechanisms and discovering potential therapeutic targets. A comprehensive search strategy was implemented to retrieve relevant articles from primary databases, including Web of Science, Google Scholar, Scopus and PubMed, for CC and metabolomics. Recent advancements in metabolomics have deepened our understanding of CC by uncovering key metabolic signatures and elucidating underlying mechanisms. By targeting crucial metabolic pathways including glucose metabolism, amino acid metabolism, fatty acid metabolism, bile acid metabolism, ketone body metabolism, steroid metabolism and mitochondrial energy metabolism, it becomes possible to restore metabolic balance and alleviate CC symptoms. This review provides a comprehensive summary of metabolomics studies in CC, focusing on the discovery of potential therapeutic targets and the evaluation of modulating specific metabolic pathways for CC treatment. By harnessing the insights derived from metabolomics, novel interventions for CC can be developed, leading to improved patient outcomes and enhanced quality of life.

## Introduction

Cancer cachexia (CC) is a complex multi‐organ and multifactorial syndrome characterized by metabolic disorders, systemic inflammation and anorexia.^1^, ^2^ It affects over 50% of cancer patients and is responsible for approximately 30% of cancer‐related deaths.^3^ CC is characterized by a prominent loss of body weight, primarily due to progressive skeletal muscle atrophy, accompanied by a possible reduction in adipose tissue mass.^4^ Skeletal muscle atrophy leads to debilitating fatigue, compromising the tolerance and efficacy of clinical anti‐cancer interventions, such as chemotherapy and surgery, and significantly impacting the patients' quality of life.^5^ Consequently, extensive research has focused on unravelling the molecular mechanisms underlying CC, with specific emphasis on skeletal muscle. The current understanding suggests that systemic inflammation and abnormal metabolism, resulting from complex tumour–host interactions, are major contributors to skeletal muscle loss in CC.

The excessive production of inflammatory cytokines, including interleukin‐6 (IL‐6), interleukin‐1 (IL‐1), tumour necrosis factor alpha (TNF‐α) and transforming growth factor beta (TGF‐β), by cancer cells and host immune cells contributes to oxidative stress, activation of the ubiquitin‐proteasome pathway (UPP) and autophagy‐lysosome pathway (ALP) and a negative nitrogen balance with increased catabolism and decreased anabolism.^6^, ^7^ These cytokines also activate downstream components, such as atrogin‐1 and muscle RING‐finger protein‐1 (MuRF1), through signalling pathways like insulin‐like growth factor 1 (IGF‐1)–protein kinase B (Akt/PKB)–forkhead box O (FoxO)/mammalian target of rapamycin (mTOR), nuclear factor kappa‐B (NF‐κB) and IL‐6–signal transducer and activator of transcription 3 (STAT3), leading to muscle atrophy and protein metabolism dysregulation. Additionally, cancer cells require a substantial amount of energy derived from peripheral organs, such as skeletal muscle, in the form of glucose, glutamine, ketone bodies and acetate,^8^, ^9^, ^10^ resulting in skeletal muscle hypercatabolism and energy deficiency.

Recent research has shown promising results in targeting specific receptors or pathways to mitigate CC, offering potential therapeutic approaches for clinical trials. For example, blocking the activin type 2 receptor (ActRIIB/ACVR2A) pathway reversed skeletal muscle atrophy and extended survival in multiple mouse models.^11^ Further research has focused on targeting specific receptors to mitigate the effects of CC. For instance, studies have shown that targeting the tumour necrosis factor receptor superfamily member 12A (Fn14/TNFRSF12A) receptor can effectively prevent inflammation and the loss of adipose and skeletal muscle mass, subsequently extending the lifespan in mouse models of CC.^12^ Additionally, targeting the receptor for advanced glycation end‐products (RAGE) has been identified as an effective strategy to reduce skeletal muscle wasting and prolong survival in CC models.^13^ More recently, studies have demonstrated the potential of activating the Akt/mTOR signalling pathway to completely reverse muscle wasting associated with CC.^14^

However, considering the metabolic abnormalities present in approximately 50% of cancer patients with cachexia, targeting metabolites and metabolic pathways could offer further anti‐cachexia therapeutic strategies.^15^ Metabolomics, an increasingly popular ‘omics’ approach, provides a comprehensive analysis of small‐molecule metabolites involved in cellular metabolism.^16^ Metabolomics, particularly through metabolic flux analysis (MFA), can capture subtle yet significant alterations in biological systems. Utilizing isotopic labels such as ^13^C and ^2^H, MFA enables a dynamic and real‐time depiction of the phenotype, thereby delivering a nuanced and thorough understanding of the intricate metabolic processes and their variations.^17^

Metabolomics has been applied to investigate various human diseases, including cancer,^18^, ^19^ coronavirus disease 2019 (COVID‐19),^20^, ^21^ sarcopenia,^22^, ^23^ cardiovascular disease^24^, ^25^ and diabetes.^26^, ^27^ In recent years, metabolomics has played a crucial role in identifying biomarkers, elucidating molecular mechanisms and discovering potential therapeutic targets.^28^ In the context of CC, metabolomics has contributed to the discovery of novel biofluid biomarkers and systemic metabolic alterations for early diagnosis and understanding the pathogenesis of CC.

In this review, we will provide a comprehensive summary of studies investigating CC using metabolomics approaches. Specifically, we will highlight the results related to the discovery of potential therapeutic targets and the evaluation of targeting specific metabolic pathways for the treatment of CC. By analysing the metabolomics data from these studies, we aim to shed light on novel strategies for managing CC and improving the outcome for cancer patients.

## Materials and methods

### Search strategy

A comprehensive search strategy was implemented to retrieve relevant articles from primary databases, including Web of Science, Google Scholar, Scopus and PubMed. The search focused on the ‘title/abstract’ field and employed advanced search techniques. The following keywords were used: ‘metabolomic and cachexia’, ‘metabolomic and muscle atrophy’, ‘metabolomics and cachexia’, ‘metabolomics and muscle atrophy’, ‘metabonomic and cachexia’, ‘metabonomic and muscle atrophy’, ‘metabonomics and cachexia’, ‘metabonomics and muscle atrophy’, ‘metabolic and cachexia’ and ‘metabolic and muscle atrophy’. The search was conducted up until May 2023 to ensure a comprehensive exploration of the available literature in this field.

### Inclusion criteria

Our review specifically focused on experimental articles investigating potential therapeutic targets for CC using the metabolomics approach. We broadened our analysis to encompass research studies that investigated the impact of metabolites or metabolic pathways on muscle atrophy, extending beyond the scope of metabolomics studies alone. We intentionally excluded articles classified as letters, reviews, editorial comments and conference abstracts from our analysis. Additionally, studies exploring cachexia induced by factors other than cancer, such as heart failure, chemotherapy, aging, chronic diseases, acquired immunodeficiency syndrome (AIDS) and disuse (immobility, bedrest and hindlimb suspension), were deliberately excluded. Furthermore, studies primarily focused on the identification of metabolic biomarkers in CC through the application of the metabolomics approach were not included in our analysis.

### Metabolomics approach

**Figure 1:**
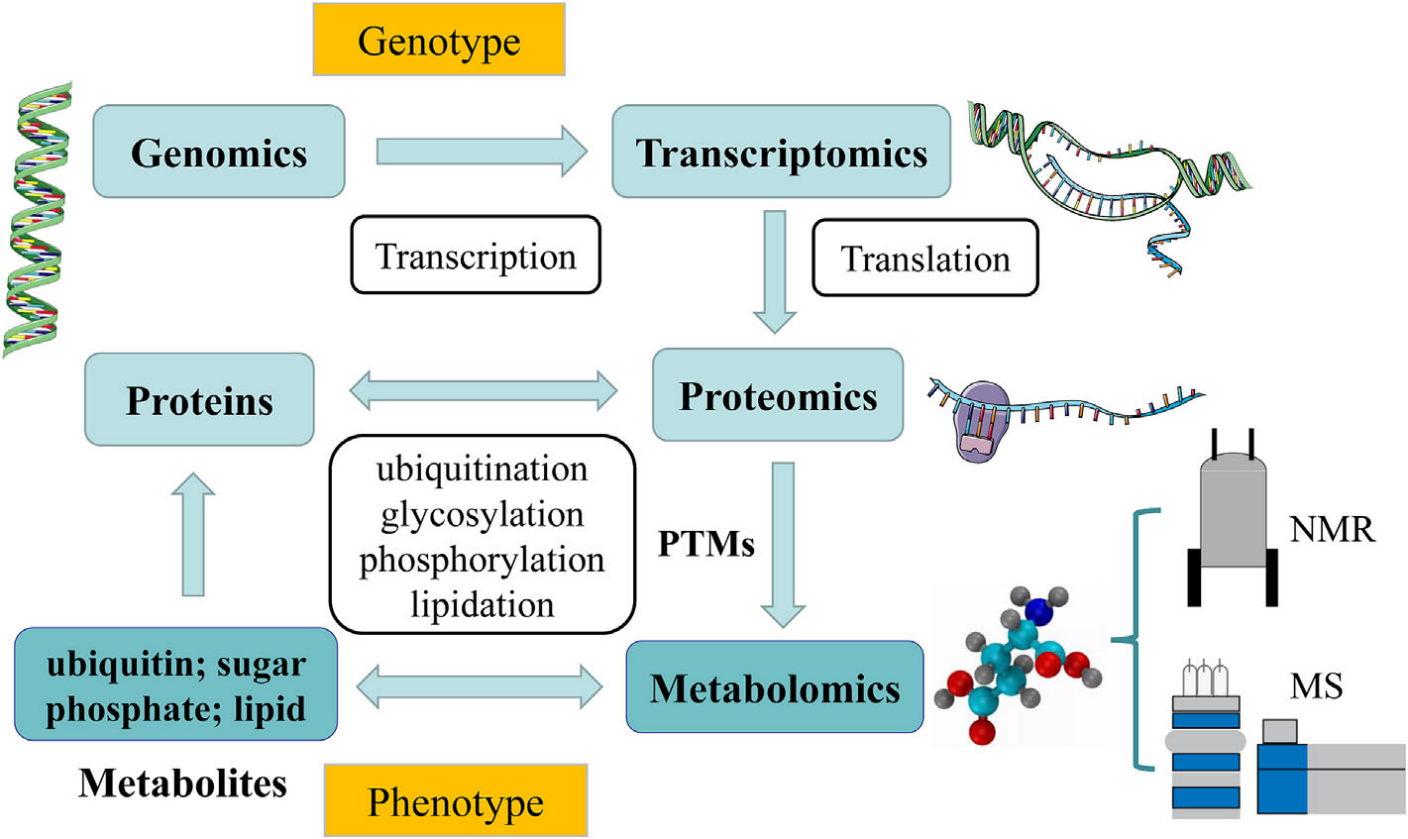
Multiple levels targeted by omics approaches in biological systems. Metabolomics can accurately and quantitatively detect downstream biological changes. MS, mass spectrometry; NMR, nuclear magnetic resonance; PTMs, post‐translational modifications.

Compared with prior ‘omics’ methodologies, such as proteomics, transcriptomics and genomics, metabolomics can accurately and quantitatively detect downstream biological changes (*Figure* *1*). The detailed procedures of metabolomics can be found in the supporting information.

## Metabolomics‐driven therapeutic targets for cancer cachexia

### Glucose metabolism

The observation of disrupted glucose metabolism in muscle atrophy suggests its significant role in the development and progression of CC.^29^, ^30^, ^31^, ^32^, ^33^ Previously, we utilized metabolomic analyses to investigate two CC murine models. We found decreased glucose levels in cachectic muscles associated with glioma,^29^ as well as reduced glucose abundance in both serum and muscle of gastric CC.^30^ Furthermore, we identified significant up‐regulation of gene expression of three key enzymes involved in the rate‐limiting steps of glucose metabolism: hexokinase, phosphofructokinase and phosphoglycerate mutase, in cachectic muscles.^30^ These results provide valuable insights into the impaired state of glucose metabolism in CC and highlight potential therapeutic targets that could be explored for intervention.

Wei et al. observed consistent results, showing a significant increase in hexokinase expression in atrophic muscles, coupled with reduced blood glucose in cachexia groups.^33^ To further investigate the potential therapeutic implications of these results, Wei et al. explored the effects of 2‐deoxy‐d‐glucose (2‐DG), a glucose analogue known to competitively inhibit hexokinase function and suppress glycolysis.^34^, ^35^ The administration of 2‐DG specifically resulted in elevated muscular glycogen levels, increased serum ketone bodies and the preservation of lean mass. Significantly, 2‐DG treatment not only stimulated liver ketogenesis and enhanced the utilization of ketone bodies by skeletal muscles but also exhibited a down‐regulatory effect on the UPP signalling and autophagy degradation.

Wei et al. revealed that 2‐DG led to significant improvements in weight loss without causing a notable reduction in tumour growth. 2‐DG has been primarily studied for its ability to disrupt tumour metabolism, specifically targeting glycolysis and adenosine triphosphate (ATP) production.^36^ These findings suggest that 2‐DG, by directly targeting muscle metabolism, may have therapeutic potential in mitigating the symptoms of CC. However, it is important to consider that the weight loss attenuation observed with 2‐DG treatment might also be partially attributed to its indirect effects on cancer metabolism. Future research should explore the dual effects of 2‐DG, examining its impact on both muscle metabolism and cancer metabolism, to fully understand its potential in combatting the multifaceted aspects of CC syndrome.

Similarly, Tseng et al. investigated the dysregulation of glucose metabolism in muscle through metabolomics analysis, revealing depleted glycogen levels and reduced abundances of glucose and key glycolytic intermediates.^37^ Additionally, they identified elevated levels of 2‐hydroxybutyrate, a biomarker associated with insulin resistance. To explore potential therapeutic strategies, the researchers explored the effects of AR‐42, a novel histone deacetylase (HDAC) inhibitor, on abnormal metabolism in CC. Their results highlighted AR‐42 as a promising therapeutic candidate for CC, emphasizing the need for further investigation into its efficacy and mechanisms of action.

In addition, Ohbuchi et al. conducted plasma metabolomics analyses to explore the effects of rikkunshito (RKT) as a traditional Japanese herbal medicine and address the underlying mechanisms.^32^ The data unveiled significant alterations in 23 metabolites following RKT treatment in CC rats. Notably, glucarate, a compound known for its anti‐cancer activity through the inhibition of β‐glucuronidase, exhibited increased levels in both the tumour‐bearing and normal groups after RKT administration. Intriguingly, the researchers investigated the therapeutic potential of glucarate in alleviating CC symptoms using a rat model. Glucarate demonstrated remarkable reductions in ascites volume, decreased levels of plasma inflammatory cytokines, improved gastrocnemius muscle atrophy and inhibited body weight loss. However, the underlying molecular mechanisms and metabolic regulations responsible for the effects of glucarate remain unknown and warrant further investigation.

In various animal models of CC, a marked decrease in glucose and glycogen levels was observed, leading to an energy deficit. This was accompanied by a reduction in metabolic intermediates and an increase in key glycolytic enzymes. Although studies have identified compounds such as 2‐DG, AR‐42 and RKT that target the impaired glucose metabolism to mitigate CC symptoms, there is a need for further research. Both preclinical models and clinical trials are essential to delve deeper into the molecular mechanisms and comprehensively evaluate the effectiveness of these drugs in treating CC.

### Amino acid metabolism

Metabolomics analyses in various animal models have consistently highlighted an imbalance between muscle protein synthesis and proteolysis as a prominent characteristic of CC.^38^, ^39^, ^40^ Notably, branched‐chain amino acids (BCAAs), which are plentiful in dietary components, are both produced and broken down by the gut microbiome. Importantly, the levels of BCAAs circulating in the host's system have been found to be associated with insulin resistance. This relationship underscores the significant impact of gut microbiome activity on host metabolism and health outcomes related to insulin sensitivity.^41^, ^42^ Der‐Torossian et al. observed a decrease in serum levels of BCAAs in CC patients compared with fasting or aging individuals.^43^ Moreover, Yang et al. conducted an integrated evaluation of serum and muscle metabolomics using high‐resolution magic angle spinning nuclear magnetic resonance (HR‐MAS NMR)‐based technology in CC mice. They observed increased ketone bodies and decreased BCAAs as the distinctive metabolic signature of CC.^31^ The gut microbiota also plays vital roles in regulating gut hormones, CC‐related cytokines and host metabolism.^44^ Furthermore, Ni et al. demonstrated the involvement of the gut microbiota in regulating gut hormones, CC‐related cytokines and host metabolism. They observed significantly reduced levels of BCAAs in the plasma of cancer patients, along with impaired BCAA functional pathways in the gut microbiota.^45^ These results collectively highlight the consistent alterations in BCAAs and their association with CC, underscoring the potential role of BCAAs and the gut microbiota in CC pathogenesis.

Individuals with CC often exhibit low levels of BCAAs, particularly leucine. Leucine plays vital roles in muscle metabolism, as it can stimulate protein synthesis through the mTOR pathway and inhibit protein breakdown.^46^, ^47^ Viana et al. demonstrated that leucine supplementation could reduce muscle loss and alter metabolic profiles without affecting tumour mass.^48^ They further found that leucine‐rich diets activated the mTOR pathway, maintained muscle function and mitigated muscle weight loss.^49^ Through the integration of metabolomics and proteomics analyses, they uncovered that the leucine‐rich diet exerted improvements in glycolysis, the tricarboxylic acid (TCA) cycle and mitochondrial function, together with the activation of protein anabolism via an increased mTOR signalling pathway.^50^ These results suggest that dietary supplementation with leucine may be a potential therapeutic strategy for CC treatment. However, it is important to conduct further research to evaluate the safety of leucine supplementation for tumour growth. A more comprehensive analysis of leucine supplementation for potential preclinical studies is discussed in other reviews.^51^, ^52^

In CC patients, skeletal muscle often exhibits an imbalance in protein catabolism and anabolism. Despite extensive research on muscle hypercatabolism, therapeutic strategies to prevent muscle atrophy in CC have had limited success. Through untargeted metabolomics analysis, Kunz et al. investigated metabolic pathways associated with muscle anabolism.^53^ The CC group showed elevated levels of asymmetric dimethylarginine (ADMA) and NG‐monomethyl‐l‐arginine, which were associated with suppressed protein synthesis. These results suggest that targeting ADMA may hold promise for enhancing muscle protein anabolism in cachexia treatment.

Through a combination of RNA sequencing and untargeted metabolomics on murine myoblasts treated with lung cancer cell‐conditioned media,^54^ Arneson‐Wissink and Doles unveiled significantly enriched pathways primarily linked to amino acid metabolism, with arginine metabolism identified as the top enriched pathway. Moreover, they demonstrated that tumour‐secreted factors modulated metabolism via nitric oxide synthase 2 (NOS2), negatively impacting myoblast differentiation. These results suggest that targeting NOS2, which regulates arginine catabolism, could potentially counteract muscle wasting induced by inflammatory cytokines. To validate and further explore these results, future studies incorporating animal models or human cohorts are warranted.

### Lipid metabolism

#### Fatty acid metabolism

Fukawa et al. investigated the underlying causes of hypermetabolism in CC and revealed an abnormality in fatty acid metabolism associated with muscle wasting.^55^ To gain insights into the metabolic signatures, they performed metabolomics analysis and demonstrated that inflammatory cytokines could induce excessive fatty acid oxidation. Interestingly, inhibition of fatty acid oxidation not only showed improvements in human myotubes in vitro but also effectively prevented weight loss in vivo. These results highlight the potential of targeting fatty acid oxidation as a promising therapeutic avenue for cachexia treatment.

Furthermore, Gumpper‐Fedus et al. reported a significant elevation in the levels of oleic acid and a corresponding decrease in the levels of linoleic acid among pancreatic cancer patients with cachexia when compared with those without cachexia.^56^ Further research is needed to evaluate the therapeutic potential of oleic acid and linoleic acid in clinical trials.

In addition to skeletal muscle and blood, metabolomics studies investigating CC have also shed light on the involvement of other tissues and organs.^57^, ^58^, ^59^, ^60^ Through metabolomics and metagenomics analyses of liver and intestinal samples from a colorectal cancer (C26) murine model of CC, Potgens et al. observed marked changes in hepatic fatty acid metabolism, including a significant increase in lipid and triglyceride levels, indicative of hepatic steatosis. Concurrently, they noted a reduction in acetate and butyrate in the intestine. Their study highlighted that decreased hepatic levels of carnitine, vital for fatty acid catabolism, and the down‐regulation of enzymes such as acyl‐CoA dehydrogenase medium chain (Acadm) and acetyl‐CoA acyltransferase 2 (Acaa2) indicated diminished mitochondrial fatty acid β‐oxidation in the liver. The lowered levels of butyrate and acetate suggest adverse effects on the symbiotic relationship between the gut microbiome and host metabolism. These comprehensive findings suggest that such disruptions in fatty acid metabolism contribute to the compromised hepatic and intestinal homeostasis observed in CC mice.^57^

#### Ketone body metabolism

Ketone bodies, including 3‐hydroxybutyrate, acetone and acetoacetate, are end‐products of fatty acid metabolism and are generated through ketogenesis in liver tissue. These molecules have been applied to various diseases due to their anti‐tumour and anti‐inflammatory properties.^61^, ^62^, ^63^ Von Renesse et al. performed serum metabolomics analyses on patients with upper gastrointestinal and pancreatic cancer and revealed that CC led to significant increases in ketone bodies, ketogenic amino acid lysine and metabolites associated with lipid utilization.^64^

We previously conducted muscle metabolomics analyses to investigate metabolic impairments using a murine model of glioma cachexia.^29^ Our results showed a significant decrease in 3‐hydroxybutyrate, a major component of ketone bodies, in cachectic muscles. Notably, 3‐hydroxybutyrate can serve as an alternative energy source to glucose during periods of energy deficiency, such as fasting. This observation suggests that ketogenic diets might be an effective treatment for CC, as they can spare glucose for tumours while providing fuel in the form of ketone bodies to skeletal muscles.

Several studies have explored the effects of ketone bodies on attenuating muscle atrophy.^63^, ^65^, ^66^ Shukla et al. performed metabolomics analyses and demonstrated that ketogenic diets induced metabolic reprogramming, including increased blood levels of ketone bodies and decreased uptake of glucose and glutamine in tumour cells. Their work showed that ketone bodies reduce tumour growth and subsequently inhibit muscle atrophy through metabolic alterations in pancreatic cancer cells and mice.^63^

Koutnik et al. have demonstrated that CC mice treated with a ketone diester exhibit systemic metabolic changes, including elevated levels of ketone bodies and decreased levels of glucose in the blood.^66^ These results suggest that ketone diester treatment can reduce muscle atrophy and exert anti‐catabolic effects by modulating the low IGF‐1/insulin signalling pathway in inflammation‐induced atrophy.

Chen et al. conducted muscle metabolomics analyses to investigate the beneficial effects of 3‐hydroxybutyrate on muscle atrophy.^65^ Their results revealed reduced purine degradation and decreased levels of uric acid, as well as an increased metabolic ratio of glutamine to glutamate in cachectic muscles. Although the used murine model was not specifically related to cancer, these results provide valuable insights into the potential role of ketone bodies in mitigating muscle wasting associated with CC.

The efficacy of ketogenic diets in cancer treatment, particularly in the context of CC, remains a topic of considerable debate. While numerous preclinical studies and a few clinical trials have shown that ketogenic diets may inhibit tumour growth, increase lifespan and mitigate the effects of CC in various animal models, contradictory findings have also been reported. These include accelerated tumour growth in some models and adverse effects like nausea, hypoglycaemia and fatigue in patients using ketone supplements. The ketogenic diet, as an adjunct cancer therapy, faces challenges due to factors like diverse tumour types, patient demographics (age, sex and general health), diet composition variations and differences in study designs. Although preclinical data often indicate beneficial impacts of ketogenic diets, clinical trial outcomes are not yet conclusive. Thus, refined research designs and specific guidelines are essential to advance the understanding and application of ketogenic diets in cancer therapy.^67^, ^68^ Considering the availability of ketone supplements, future research should concentrate on diverse cancer types and evaluate the long‐term implications of exogenous ketones in human trials, particularly focusing on cancer progression and risk.

#### Bile acid metabolism

Furthermore, Thibaut et al. identified altered metabolites associated with bile acid metabolism and further investigated the role of bile acid pathways in liver inflammation and CC.^58^ Similarly, Feng et al. performed metabolomic profiling to elucidate the involvement of the liver and intestinal microbes in impaired bile acid metabolism in the C26 model, revealing up‐regulated bile acid conjugation, down‐regulated bile acid synthesis and microbial bile acid metabolism in CC mice.^59^ These studies highlight the importance of investigating metabolic alterations in different organs and their potential contribution to the pathogenesis of CC.

Feng et al. investigated the potential therapeutic effects of tauroursodeoxycholic acid on cachexia by targeting bile acid metabolism. Surprisingly, this agent demonstrated significant efficacy in reducing symptoms associated with CC, including muscle, heart and liver atrophy and weight loss. Based on these results, Thibaut et al. explored the potential of another agent, ursodeoxycholic acid, for CC treatment^60^; however, their results indicated that this agent neither reduced liver inflammation nor rescued muscle atrophy in CC mice. These contrasting results highlight the complexity of bile acid metabolism in CC and the need for further research in this area.

Takeda G protein‐coupled receptor 5 (TGR5) has also gained attention as a promising therapeutic target for CC due to its involvement in bile acid activation.^60^ These studies suggest that interventions targeting bile acid metabolism, such as the use of tauroursodeoxycholic acid or TGR5 agonists, may hold potential as beneficial strategies for CC treatment. Expectedly, interventions aimed at improving treatment outcomes for CC may increasingly focus on understanding and targeting the metabolism of skeletal muscle, liver and gut microbes. By unravelling the intricate interplay between these organs and their metabolic alterations in CC, novel therapeutic avenues may be identified to effectively manage this complex syndrome.

#### Steroid metabolism

Steroids, synthesized in small amounts from cholesterol, are complex lipophilic biomolecules with chemical properties similar to lipids.^69^ These metabolites, including cortisol, aldosterone, oestradiol and testosterone, play critical roles in regulating metabolism, sexual development and reducing inflammation.^70^ Steroids have been traditionally used in both preclinical models and human trials to treat cachexia.

Specifically, corticosteroids like dexamethasone can induce myotube atrophy at certain doses by increasing MuRF1 expression.^71^ A recent study by Ferrer et al. demonstrated that dexamethasone treatment increased food intake, reduced hepatic fatty acid metabolism, delayed cachexia progression and prolonged lifespan in CC mice on ketogenic diets.^72^ While corticosteroids are known to improve appetite and body weight and are beneficial in CC management,^73^ their use is typically limited to less than a month due to potential side effects and decreased efficacy over time.^74^

Glucocorticoids, another class of steroids, play vital metabolic roles. They have been found in increased concentrations in both murine models and patients with CC,^75^ known to up‐regulate protein degradation and down‐regulate synthesis in skeletal muscles.^76^ However, a glucocorticoid receptor antagonist did not reverse CC in a murine model,^77^ suggesting the need for further studies to understand glucocorticoids' role in CC. Consequently, the activation of glucocorticoid‐responsive genes plays a significant role in skeletal muscle protein catabolism and liver metabolism.^78^ This involvement is particularly critical in the context of CC, where these genes contribute to the metabolic alterations observed in affected tissues. However, the precise role of glucocorticoids in the pathophysiology of CC remains to be fully elucidated. Therefore, further research, both in preclinical models and in clinical trials, is essential to deepening our understanding of the impact of glucocorticoids on CC, ultimately leading to more effective therapeutic strategies.

Decreased levels of circulating testosterone, linked to hypogonadism in CC patients, have been observed.^79^ Testosterone administration can increase muscle growth and lean body mass.^80^ Testosterone analogues like oxandrolone have shown benefits in cachexia treatment but require careful monitoring due to potential side effects.^15^, ^81^, ^82^ In summary, the use of steroid hormones in the treatment of CC shows potential as an effective therapeutic strategy. However, it is crucial to meticulously weigh the advantages and disadvantages of steroid use in clinical trials. This careful consideration is necessary to ensure that the therapeutic benefits of steroids significantly outweigh any potential risks associated with their use. Such an approach is essential for optimizing patient outcomes and minimizing adverse effects in the management of CC.

In various preclinical and clinical models of CC, notable metabolic alterations have been observed, including decreased levels of ketone bodies and circulating testosterone, diminished bile acid synthesis and increased fatty acid oxidation. To address these changes, recent research has focused on molecules like tauroursodeoxycholic acid, TGR5 agonists, ketone esters and steroid hormones that target lipid metabolism, aiming to mitigate the symptoms of CC. Among the potential treatments, ketogenic diets and steroid‐based therapies are emerging as promising approaches. The availability of ketone supplements and steroid analogues on the market underscores the need for future research. This research should concentrate on exploring the efficacy of these treatments across various cancer types, assessing the associated cancer risks and monitoring disease progression during long‐term effects in human trials.

### Mitochondrial energy metabolism

In mammalian cells, acetyl‐CoA is typically generated within mitochondria through the decarboxylation of pyruvate, β‐oxidation of fatty acids and catabolism of amino acids.^83^ Crucially, disturbances in glucose, amino acid and lipid metabolism might lead to compromised oxidative phosphorylation and mitochondrial dysfunction.^84^ CC is a metabolic derangement associated with impaired mitochondrial function and disturbed energy metabolism, which is induced by muscle protein imbalance and an inflammatory response.^85^, ^86^, ^87^, ^88^ Pin et al. performed comprehensive metabolomics analyses to characterize the metabolic signatures between chemotherapy‐induced cachexia and CC.^89^ Metabolomics analysis revealed a significant decrease in plasma glucose, citrate and succinate. Muscle metabolic profiling further demonstrated a decrease in TCA cycle flux, accompanied by down‐regulated enzymatic activities of pyruvate dehydrogenase (PDH) and succinate dehydrogenase (SDH). Additionally, the levels of succinate and the energy carriers ATP and creatine phosphate were found to be significantly reduced. Thus, cachectic muscles show down‐regulated oxidative phosphorylation, impaired TCA cycle activity and an intracellular energy deficiency, collectively resulting in mitochondrial dysfunction.

### Metabolic regulators by compounds

Researchers have used several compounds, such as SS‐31, trimetazidine (TMZ), curcumin and niacin, that can all act as metabolic regulators to regulate mitochondrial function and improve impaired mitochondrial metabolism.

#### Metabolic alterations induced by SS‐31 treatment

Ballaro et al. undertook a study with the objective of targeting mitochondrial dysfunction as a strategic approach to combat muscle wasting in CC.^90^ They utilized SS‐31, a mitochondria‐targeted peptide that binds to cardiolipin on the inner mitochondrial membrane,^91^, ^92^, ^93^ to protect and preserve mitochondrial integrity. Using the C26 model, they examined the impact of SS‐31 on muscle loss. Additionally, they conducted metabolomic analyses of plasma, liver and skeletal muscle to elucidate the metabolic alterations with SS‐31 treatment. The results revealed that SS‐31 had a partial regulatory effect on liver and muscle metabolism. Although SS‐31 did not prevent mitochondrial protein loss or promote protein anabolism, it did improve muscle SDH activity, including increased levels of intracellular ATP, alanine, glucose, glycogen and glutamine. These results underscore the significance of maintaining mitochondrial function and achieving a balance in energy metabolism in muscle and other organs for future CC treatments. The study highlights the interconnectedness of metabolism across multiple organs and emphasizes the importance of targeting mitochondrial dysfunction as a potential therapeutic approach for CC.

#### Metabolic alterations induced by trimetazidine treatment

In recent research, TMZ, a metabolic modulator, has shown promise in counteracting muscle atrophy by improving mitochondrial function and correcting abnormal metabolism.^94^, ^95^, ^96^ To investigate its potential therapeutic effects, Molinari et al. conducted a study using the C26 model.^97^ Although TMZ was unable to delay the loss of body weight or the weight of specific muscles such as the tibialis anterior and gastrocnemius, it did have positive effects on metabolic modulations such as increased grip force, stimulated mitochondrial biogenesis and improved energy metabolism. Specifically, the expressions of key regulators such as peroxisome proliferator‐activated receptor gamma coactivator 1‐alpha (PGC1α), transcription factor A, protein Tom20 and ATP synthase were partially restored, and the phosphoinositide 3‐kinase (PI3K)/AKT signalling pathway was involved in the maintenance of muscle mass. Furthermore, TMZ treatment effectively increased SDH activity in vivo and enhanced mitochondrial membrane potential in vitro.

Conclusively, it is noteworthy that TMZ is a clinically approved drug and possesses exercise‐mimetic effects, which suggests that it could be beneficial for individuals with CC who are unable to engage in physical activity. The results of this study highlight the potential of TMZ as a therapeutic option for improving mitochondrial function and metabolism in the context of CC.

#### Metabolic alterations induced by curcumin treatment

Curcumin, a traditional Asian herb with diverse bioactivities, including anti‐tumour and anti‐inflammatory properties,^98^, ^99^ has attracted attention for mitigating muscle atrophy. Recently, Zhang et al. conducted a study using muscle metabolomic analyses and biochemical experiments to investigate the effects of curcumin on muscle atrophy in a murine model.^100^

The results showed that curcumin treatment delayed the loss of body weight and skeletal muscle mass, restored muscle force strength and muscle fibre density and improved mitochondrial morphology and ATP generation by inhibiting the activation of the NF‐κB/UPP signalling pathway. Muscle metabolomics analysis demonstrated that curcumin modulated energy metabolism associated with glucose metabolism, pyruvate metabolism, the TCA cycle and glyoxylate and dicarboxylate metabolism, thus alleviating muscle loss.

Similarly, Penedo‐Vázquez et al. demonstrated that curcumin treatment effectively delayed the signalling pathways associated with muscle atrophy and proteolysis in CC mice. Moreover, curcumin treatment promptly improved the condition of type I and type II limb muscle fibres, surpassing the outcomes observed in untreated cachexia mice.^101^ Chaiworramukkul et al. investigated the effects of curcumin in CC patients.^102^ Although no statistically significant differences were observed in body composition parameters such as muscle and adipose tissue mass between the curcumin and placebo groups, patients receiving curcumin exhibited diminished handgrip muscle force loss and basal metabolic rate compared with those receiving placebo.

Overall, these studies provide encouraging evidence for the potential benefits of curcumin in mitigating muscle atrophy and improving muscle function in the context of CC. However, further investigations with larger cohorts of cancer patients and optimized clinical assessment of curcumin are necessary to fully understand its therapeutic effects and establish its clinical applications.

#### Metabolic alterations induced by niacin treatment

Recently, Beltra et al. performed metabolomics analyses in human muscle biopsies and serum based on the expression of the nicotinamide adenine dinucleotide (NAD^+^) biosynthetic enzyme nicotinamide riboside kinase 2 (NRK2).^103^ Compared with healthy and high NRK2 muscles, low NRK2 muscles displayed increased amounts of glycolysis and TCA intermediates, nucleotides and amino acids, representing the outcome of disturbed energy metabolism. To counteract the CC syndromes induced by deficiency of NAD^+^ and down‐regulation of NRK2, the researchers explored the therapeutic effects of a NAD^+^ precursor niacin in a CC mouse model. Surprisingly, vitamin B3 niacin significantly restored the NAD^+^ abundances in muscle and liver, improved mitochondrial biogenesis and attenuated CC symptoms. Overall, their results imply NAD^+^ and mitochondrial metabolism as promising therapeutic targets and highlight the development of niacin‐based treatments for CC patients in the future.

## Conclusions and perspectives

This review provides a comprehensive summary of metabolomics studies focusing on the discovery of potential therapeutic targets for CC and the evaluation of treatment outcomes targeting specific metabolic pathways in the past decade (*Table* *1*). Notably, interventions aimed at modulating these pathways, such as glucose, amino acid, fatty acid, bile acid, ketone body, steroid and mitochondrial energy metabolism, have demonstrated promising effects in restoring muscle mass and function, attenuating body weight loss and extending lifespan in CC models. Importantly, these studies have revealed the interplay and cross‐talk among various tissues and organs involved in CC. For example, hepatic gluconeogenesis and the Cori cycle, which involve muscle glycolysis, and the interaction between bile acid metabolism and gut microbial activities play roles in the enterohepatic cycle. Moreover, ketone body metabolism is known to affect hepatic fatty acid oxidation and the TCA cycle in muscle mitochondria. These interconnected metabolic processes emphasize the complex nature of CC and the need for a systemic approach to its treatment.

**Table 1:** Summary of metabolomics‐driven discoveries for cancer cachexia

Reference	Study object	Altered metabolic pathway	Identified targets	Potential strategy
Shukla et al. (2014)^63^	Mice	Fatty acid metabolism	Ketone bodies	Ketogenic diet
Ohbuchi et al. (2015)^32^	Rats	Glucose metabolism	Glucarate	Rikkunshito
Tseng et al. (2015)^37^	Rats	Glucose metabolism	Glycolysis; glycogen synthesis	AR‐42
Fukawa et al. (2016)^55^	Mice	Fatty acid metabolism	Fatty acid oxidation	Etomoxir
Viana et al. (2016)^48^	Rats	Amino acid metabolism	BCAAs	Leucine
Molinari et al. (2017)^97^	Mice	Mitochondrial energy metabolism	Mitochondrial succinate dehydrogenase	Trimetazidine
Cui et al. (2019)^30^	Mice	Glucose metabolism	Hexokinase; phosphofructokinase; phosphoglycerate mutase	
Koutnik et al. (2020)^66^	Mice	Fatty acid metabolism	Ketone bodies	Ketone diester
Cruz et al. (2020)^50^	Rats	Amino acid metabolism	BCAAs	Leucine
Kunz et al. (2020)^53^	Mice/C2C12 cell	Amino acid metabolism	Methylarginine	Asymmetric dimethylarginine
Ballaro et al. (2021)^90^	Mice	Mitochondrial energy metabolism	Mitochondrial succinate dehydrogenase	SS‐31
Arneson‐Wissink and Doles (2021)^54^	C2C12 cell	Amino acid metabolism	Nitric oxide synthase 2	1400W
Potgens et al. (2021)^57^	Mice	Glucose metabolism	Glycolysis	
Feng et al. (2021)^59^	Mice	Fatty acid metabolism	Bile acid metabolism	Tauroursodeoxycholic acid
Von Renesse et al. (2023)^64^	Patients	Fatty acid metabolism	Ketone bodies; ketogenic amino acid lysine	
Gumpper‐Fedus et al. (2022)^56^	Patients	Fatty acid metabolism	Oleic acid; linoleic acid	
Zhang et al. (2022)^100^	Mice	Mitochondrial energy metabolism	Mitochondrial morphology; ATP	Curcumin
Wei et al. (2022)^33^	Mice	Glucose metabolism	Hexokinase	2‐Deoxy‐d‐glucose
Ferrer et al. (2023)^72^	Mice	Fatty acid metabolism	Fatty acid metabolites	Dexamethasone + ketogenic diets
Beltra et al. (2023)^103^	Patients Mice	Mitochondrial energy metabolism	NAD^+^	Niacin

Abbreviations: ATP, adenosine triphosphate; BCAAs, branched‐chain amino acids; NAD^+^, nicotinamide adenine dinucleotide.

Significant advances have been made in preclinical research on potential therapies for CC, yet translating these therapies into clinical practice remains challenging. The primary obstacle is the inability of animal models to fully represent the complex metabolic syndrome observed in CC patients. Different animal models offer varied benefits and limitations, and the current therapeutic approaches do not address all aspects of the CC syndrome effectively.

Human CC is a multifaceted, heterogeneous condition with varying levels of inflammation, anorexia, metabolic disturbances, muscle loss and metastasis. However, current animal models fall short of fully representing these diverse clinical aspects.^104^, ^105^ Particularly in advanced lung cancers, anorexia emerges as a critical symptom of CC, leading to reduced food intake and exacerbating systemic metabolic issues. Unlike metabolic changes caused by starvation or malabsorption, cancer‐induced anorexia presents unique challenges. Most standard animal models for CC do not adequately mimic this aspect,^106^ which hampers research into treatments aimed at improving food intake in cachexia. This gap in representation also complicates our understanding of whether the metabolic alterations observed in CC are consequences of, or inherently connected to, the anorexia driven by the cancer itself.

The complex interplay between cancer‐induced anorexia and metabolic alterations in CC can be understood as a bidirectional relationship with three key aspects:

- Anorexia has effects on metabolism: Anorexia can obviously reduce food intake and significantly exacerbate the metabolic imbalances inherent in cachexia. This leads to a metabolic shift towards catabolism, where the body relies increasingly on its muscle and fat stores for energy. This catabolic state further accelerates the progression of cachexia by increasing muscle wasting and weight loss.
- Metabolic changes affect appetite: The metabolic abnormalities associated with cachexia can profoundly affect the central nervous system and gastrointestinal system. These changes disrupt the normal balance of appetite‐regulating hormones and neural pathways, potentially reducing appetite and exacerbating anorexia. This demonstrates how metabolic changes in cachexia can, in turn, exacerbate anorexia.
- Psychological factors play roles in cachexia: The psychological burden of a cancer diagnosis and associated stress can significantly affect both metabolism and appetite. Stress and emotional distress can suppress appetite while affecting metabolic processes, often exacerbating cachexia symptoms. This creates a cyclical feedback loop in which psychological factors exacerbate both anorexia and metabolic dysregulation in cachexia.

This bidirectional relationship underscores the complexity of CC and the need for comprehensive treatment strategies that address both the physical and psychological aspects of the disease. Effective treatment of CC requires multimodal approaches that address both the direct metabolic effects of cachexia and the contributing role of anorexia. These include nutritional support, pharmacological interventions and psychological therapies.

Given the complexity and heterogeneity of CC, current animal models fall short of mirroring the intricate metabolic syndrome seen in CC humans. Furthermore, potential therapies, including those not driven by metabolomics, often fail to alleviate most CC symptoms. For better translation into clinical applications, it is essential to choose or develop animal models that closely match the specific characteristics of CC. Utilizing multiple CC models for research and adopting multimodal approaches to therapy are recommended strategies.

CC presents as a complex syndrome affecting multiple organs, posing considerable challenges in its treatment. Despite extensive research, no drugs have been approved in Europe or the United States specifically to improve functional parameters and body composition in CC patients. Historically, treatments have included human growth hormone for AIDS‐related cachexia in the United States and megestrol acetate for cachexia and anorexia syndrome, although its use is restricted to Korea. In 2020, the American Society of Clinical Oncology (ASCO) guidelines suggested corticosteroids and progesterone analogues to enhance appetite and body weight in CC patients.^74^ However, the use of corticosteroids may result in adverse side effects and is recommended only in low doses and for short periods. Progestins have not been shown to significantly increase lean body mass.^107^

In a significant development, Japan approved anamorelin in 2020 to treat CC, marking the first such approval for this condition. Anamorelin, a ghrelin mimetic agonist, has demonstrated efficacy in improving anorexia and increasing muscle mass and body weight in CC patients. However, it does not appear to improve muscle strength or life expectancy and is associated with side effects like nausea and constipation.^108^

**Figure 2:**
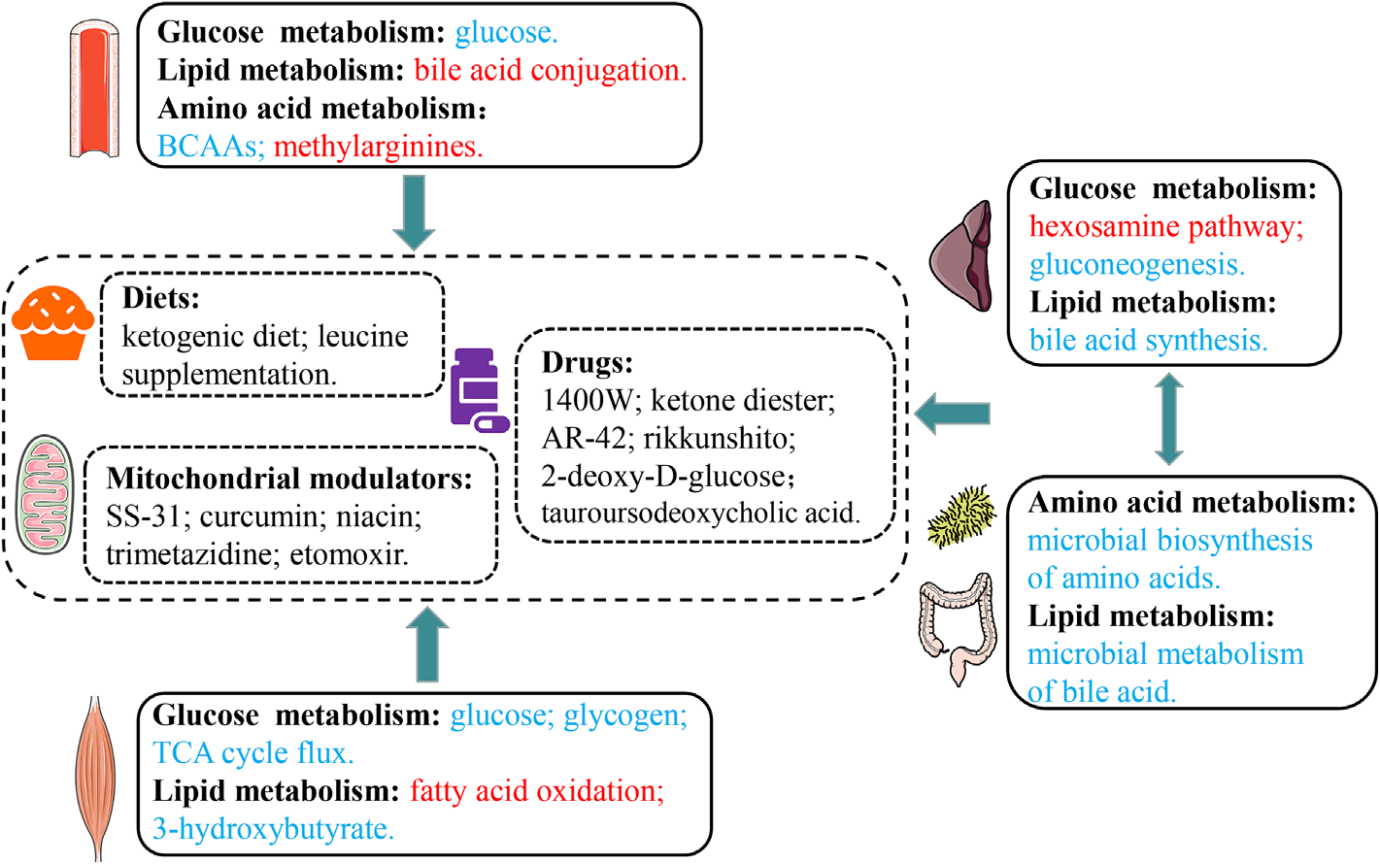
Overview of potential therapeutic targets discovered through metabolomics studies and their corresponding evaluation in cancer cachexia (CC). Up‐regulated metabolites/pathways in the CC group compared with the normal control group are represented in red, while down‐regulated metabolites/pathways are shown in blue. These findings offer insights into the molecular alterations associated with CC and highlight potential targets for therapeutic interventions. BCAAs, branched‐chain amino acids; TCA, tricarboxylic acid.

Metabolomics has emerged as a powerful tool in CC treatment, allowing for the precise identification of metabolic alterations and the development of personalized treatments. By targeting specific metabolic pathways, it is possible to restore metabolic homeostasis and alleviate CC symptoms. This approach includes the use of metabolites like BCAAs, ketone bodies and ketone diesters as nutritional supplements. Additionally, agents such as traditional herbal medicines (e.g., RKT and curcumin), clinically approved drugs (e.g., TMZ, tauroursodeoxycholic acid and ursodeoxycholic acid), peptides (e.g., SS‐31) and common chemical compounds (e.g., niacin) are being explored (*Figure* *2*). These diverse interventions offer promising avenues for developing effective therapeutic strategies against CC.

For future clinical treatments, a multidisciplinary approach that combines pharmacological therapy, exercise and nutritional intervention is strongly recommended. This holistic approach aligns with international guidelines for managing CC patients. Metabolomics‐driven potential targets, such as BCAAs, ketogenic diets and clinically approved drugs like TMZ, have shown beneficial effects on skeletal muscle metabolism. These may act as adjuncts to nutritional interventions and exercise, complementing existing therapies like anamorelin in human trials.

## Conflict of interest statement

The authors declare no conflict of interest.

## Funding

This work was supported by the Scientific Research Foundation of Xuchang University (No. 962005), the Education Department of Henan Province (No. 24A550016) and the National Natural Science Foundation of China (Nos 31971357 and 32171174).

## Supporting information


**Data S1.** Supporting Information.
