# Supplementary material for: Metabolomics‐driven discovery of therapeutic targets for cancer cachexia
Source: J Cachexia Sarcopenia Muscle. 2024 Apr 21;15(3):781–93. doi: 10.1002/jcsm.13465 (PMC11154780; doi:10.1002/jcsm.13465)
Supplement: Supplementary file 1 — Data S1. Supporting Information. [file JCSM-15-781-s001.docx]

**Supplemental materials**

***2.3. Metabolomics Approach***

Omics is an interdisciplinary methodology encompassing genomics, transcriptomics, proteomics, and metabolomics, which are rapidly advancing fields in biomedicine [1-3]. Metabolomics is centered around the study of metabolites with relatively low molecular weights (≤1500 Daltons), which are the end products of cellular processes [4]. By analyzing the metabolome, metabolomics directly captures downstream alterations and effectively reflects the phenotype [5, 6]. **Metabolomics is often viewed as a natural extension of proteomics, given that the majority of metabolites are produced through the action of enzymes, which are themselves proteins. These metabolites serve various roles, including acting as precursors and signaling molecules. They exert influence by regulating post-translational modifications (PTMs) of proteins and affecting the structure and function of chromatin. Key types of PTMs include ubiquitination, glycosylation, phosphorylation, and lipidation. These modifications are facilitated by specific metabolites like ubiquitin, various sugars, phosphates, and lipids. Additionally, PTMs have the capacity to influence enzyme functions through mechanisms such as substrate or cofactor binding, promoting degradation, influencing oligomerization, and determining cellular localization. In essence, the interplay between proteomics and metabolomics can be encapsulated in the paradigm: “Metabolites regulate PTMs, PTMs regulate enzymes, and enzymes modulate metabolites.” [7].**

Metabolomics primarily focuses on the analysis of organic acids, peptides, sugars, amino acids, and lipids, among other metabolite classes [8-11]. By simultaneously identifying hundreds to thousands of compounds, metabolomics provides a comprehensive view of the metabolites present in various sample types, including cells, tissues, biofluids, and gases, thereby offering valuable insights into cellular metabolism. Over the years, the rapid advancements in analytical instruments, acquisition software, and computational analysis tools have significantly expanded the applications of metabolomics in diverse scientific disciplines [12].

Metabolomics analysis can be conducted using two main approaches: untargeted and targeted metabolomics. Among these, untargeted metabolomics is a widely employed tool in studies related to CC. The workflow of untargeted metabolomics analysis involves several essential steps, including sample pre-treatment, utilization of analytical equipment, data acquisition, data preprocessing and analysis, metabolite identification, and data interpretation. Comprehensive reviews provide detailed explanations of these intricate processes, offering extensive insights into the field of metabolomics analysis [12-16].

In CC studies, metabolomics analysis utilizes a wide range of sample types, including skeletal muscle, blood, urine, tumor tissue, liver, brain, kidney, and gut microbiota. Proper collection and freezing of samples are crucial in the pre-treatment process to preserve the integrity of metabolites. Data acquisition is typically performed using two main analytical techniques in metabolomics: nuclear magnetic resonance (NMR)-based and mass spectrometry (MS)-based platforms (Figure 1). **NMR spectroscopy is a powerful tool in metabolomics due to its ability to detect metabolites based on their unique resonance frequencies. This technique is highly effective in identifying and quantifying metabolites in a sample, providing insights into their molecular structures. On the other hand, MS spectroscopy is utilized for its precision in detecting metabolites according to their mass-to-charge ratios (m/z). MS is particularly useful for its high sensitivity and specificity, allowing it to be an essential tool for identifying and quantifying a wide range of metabolites in complex biological samples.**

While NMR-based and MS-based methods have their respective strengths and weaknesses [17], studies have demonstrated the advantages of using a multi-platform metabolomic analysis, which offers excellent complementarity [18-21]. This approach enhances metabolite coverage and improves the reliability and robustness of the results.

**Recent advancements in in situ “-omics” techniques, particularly Mass Spectrometry Imaging (MSI), have revolutionized the study of cachexia, particularly in muscle tissue analysis [22]. MSI enables the identification of a wide spectrum of metabolic alterations in muscle tissues, providing a comprehensive view of the metabolic changes associated with cachexia. This method offers a unique advantage as it allows direct analysis of skeletal muscle tissues obtained from clinical cachexia patients. By using MSI, researchers can delve deeper into the complex metabolic networks and pathways altered in cachexia, significantly enhancing the scope and depth of metabolomics research. This enhanced capability to detect and analyze metabolic changes directly in tissue samples is pivotal for advancing our understanding of cachexia at the molecular level.**

Data preprocessing and analysis are essential steps in metabolomics analysis. They involve procedures such as data normalization, scaling, multivariate statistical analysis, and univariate analysis. Various databases and software tools are widely utilized for data analysis purposes. Classical databases such as **human metabolome database** (HMDB), **small molecule pathway database** (SMPDB), Lipid maps database, and **kyoto encyclopedia of genes and genomes** (KEGG), along with software tools like Chenomx NMR Suite, **soft independent modeling class analog** (SIMCA), Biocrates MetIDQ, and MetaboAnalyst 5.0, are commonly used in metabolomics studies for data interpretation and analysis. These resources provide valuable support for metabolite identification, pathway analysis, and the exploration of metabolomic data in the context of CC research [23, 24].

While comprehensive reviews on omics studies related to CC have been published in recent years [15, 25-27], this section of the literature aims to provide a concise overview of metabolomics studies specifically focused on discovering potential therapeutic targets for CC. Furthermore, it examines the outcomes of interventions targeting specific metabolic pathways as potential strategies for the treatment of CC, thereby assessing their effectiveness in mitigating the impact of the condition.

CC is a complex metabolic syndrome characterized by the progressive loss of skeletal muscle and adipose tissue, leading to significant weight loss in affected individuals. Metabolomics has shown great promise in unraveling the metabolic alterations associated with CC. Metabolomics researchers have been able to identify unique metabolic signatures that are closely linked to glucose metabolism, amino acid metabolism , and lipid metabolism, all of which play crucial roles in energy metabolism. In this review, we provide a comprehensive overview of studies that have utilized the metabolomics approach to discover potential therapeutic targets for CC. These studies have not only illuminated the molecular mechanisms underlying CC but have also investigated the effects of specific metabolites and the modulation of distinct metabolic pathways as potential treatment strategies (Figure 2). By highlighting these results of the studies, we seek to deepen our understanding of CC pathogenesis and provide valuable insights into novel therapeutic interventions.

**Abbreviations: Acaa2, Acetyl-CoA acyltransferase 2; Acadm, Acyl-CoA dehydrogenase medium chain; ActRⅡB/ACVR2A, Activin type 2 receptor; ADMA, Asymmetric dimethylarginine; AIDS, Acquired immunodeficiency syndrome; ALP, Autophagy lysosome pathway; Akt/PKB, Protein kinase B; ASCO, American society of clinical oncology; ATP, Adenosine triphosphate; BCAAs, Branched-chain amino acids; CC, Cancer cachexia; C26, C26 colorectal cancer; COVID-19, Coronavirus disease 2019; Foxo, Forkhead box O; Fn14/ TNFRSF12A, Tumor necrosis factor receptor superfamily member 12A; HR-MAS NMR, High-resolution magic angle spinning nuclear magnetic resonance; HDAC, Histone deacetylases; HMDB, Human metabolome database; IGF-1, Insulin-like growth factor 1; IL-1, Interleukin-1; IL-6, Interleukin-6; KEGG, Kyoto encyclopedia of genes and genomes; LLC, Lewis lung cancer; MFA, Metabolic flux analysis; MS, Mass spectrometry; MSI, Mass spectrometry imaging; mTOR, Mammalian target of rapamycin; MuRF1-Muscle RING-finger protein-1; NAD, Nicotinamide adenine dinucleotide; NF-κB, Nuclear factor kappa-B; NMR-Nuclear magnetic resonance; NOS2, Nitric oxide synthase 2; NRK2, Nicotinamide riboside kinase 2; PDH, Pyruvate dehydrogenase; PGC-1α, Peroxisome proliferator-activated receptor-γ coactivator; PI3K, Phosphoinositide 3-kinase; PTMs, Post-translational modifications; RAGE, Receptor of advanced glycation end products; RKT, Rikkunshito; SDH, Succinate dehydrogenase; SIMCA, Soft independent modeling class analog; SMPDB, Small molecule pathway database; STAT3, Signal transducer and activator of transcription 3; TCA, Tricarboxylic acid; TNF-α, Tumor necrosis factor alpha; TGF-β, Transforming growth factor beta; TGR5, Takeda G protein-coupled receptor 5; TMZ, Trimetazidine; UPP, Ubiquitin proteasome pathway.**

**References**

1. Yamada R, Okada D, Wang J, Basak T, Koyama S. Interpretation of omics data analyses. J Hum Genet. 2021;66:93-102.

2. Hasin Y, Seldin M, Lusis A. Multi-omics approaches to disease. Genome Biol. 2017;18:83.

3. Coughlin SS. Toward a road map for global -omics: a primer on -omic technologies. Am J Epidemiol. 2014;180:1188-1195.

4. Alldritt I, Greenhaff PL, Wilkinson DJ. Metabolomics as an Important Tool for Determining the Mechanisms of Human Skeletal Muscle Deconditioning. Int J Mol Sci. 2021;22:13575.

5. Griffin JL, Shockcor JP. Metabolic profiles of cancer cells. Nat Rev Cancer. 2004;4:551-561.

6. Bujak R, Struck-Lewicka W, Markuszewski MJ, Kaliszan R. Metabolomics for laboratory diagnostics. J Pharm Biomed Anal. 2015;113:108-120.

7. Nalbantoglu S, Karadag A. Metabolomics bridging proteomics along metabolites/oncometabolites and protein modifications: Paving the way toward integrative multiomics. J Pharm Biomed Anal. 2021;199:114031.

8. Fiehn O, Kopka J, Dormann P, Altmann T, Trethewey RN, Willmitzer L. Metabolite profiling for plant functional genomics. Nat Biotechnol. 2000;18:1157-1161.

9. Nicholson JK, Lindon JC, Holmes E. 'Metabonomics': understanding the metabolic responses of living systems to pathophysiological stimuli via multivariate statistical analysis of biological NMR spectroscopic data. Xenobiotica. 1999;29:1181-1189.

10. Wishart DS, Jewison T, Guo AC, Wilson M, Knox C, Liu Y, et al. HMDB 3.0--The Human Metabolome Database in 2013. Nucleic Acids Res. 2013;41:D801-807.

11. Oliver SG, Winson MK, Kell DB, Baganz F. Systematic functional analysis of the yeast genome. Trends Biotechnol. 1998;16:373-378.

12. Wishart DS. Emerging applications of metabolomics in drug discovery and precision medicine. Nat Rev Drug Discov. 2016;15:473-484.

13. Schmidt DR, Patel R, Kirsch DG, Lewis CA, Vander Heiden MG, Locasale JW. Metabolomics in cancer research and emerging applications in clinical oncology. CA Cancer J Clin. 2021;71:333-358.

14. Beckonert O, Keun HC, Ebbels TM, Bundy J, Holmes E, Lindon JC, et al. Metabolic profiling, metabolomic and metabonomic procedures for NMR spectroscopy of urine, plasma, serum and tissue extracts. Nat Protoc. 2007;2:2692-2703.

15. Cui P, Li X, Huang C, Li Q, Lin D. Metabolomics and its Applications in Cancer Cachexia. Front Mol Biosci. 2022;9:789889.

16. Dang VT, Huang A, Werstuck GH. Untargeted Metabolomics in the Discovery of Novel Biomarkers and Therapeutic Targets for Atherosclerotic Cardiovascular Diseases. Cardiovasc Hematol Disord Drug Targets. 2018;18:166-175.

17. Emwas AH. The strengths and weaknesses of NMR spectroscopy and mass spectrometry with particular focus on metabolomics research. Methods Mol Biol. 2015;1277:161-193.

18. Ahmad Azam A, Ismail IS, Shaikh MF, Abas F, Shaari K. Multi-Platform Metabolomics Analyses Revealed the Complexity of Serum Metabolites in LPS-Induced Neuroinflammed Rats Treated with Clinacanthus nutans Aqueous Extract. Front Pharmacol. 2021;12:629561.

19. Bouatra S, Aziat F, Mandal R, Guo AC, Wilson MR, Knox C, et al. The human urine metabolome. PLoS One. 2013;8:e73076.

20. Psychogios N, Hau DD, Peng J, Guo AC, Mandal R, Bouatra S, et al. The human serum metabolome. PLoS One. 2011;6:e16957.

21. Pin F, Barreto R, Couch ME, Bonetto A, O'Connell TM. Cachexia induced by cancer and chemotherapy yield distinct perturbations to energy metabolism. J Cachexia Sarcopenia Muscle. 2019;10:140-154.

22. Kunzke T, Buck A, Prade VM, Feuchtinger A, Prokopchuk O, Martignoni ME, et al. Derangements of amino acids in cachectic skeletal muscle are caused by mitochondrial dysfunction. Journal of Cachexia, Sarcopenia and Muscle. 2019;11:226-240.

23. Pang Z, Zhou G, Ewald J, Chang L, Hacariz O, Basu N, et al. Using MetaboAnalyst 5.0 for LC-HRMS spectra processing, multi-omics integration and covariate adjustment of global metabolomics data. Nat Protoc. 2022;17:1735-1761.

24. Pang Z, Chong J, Zhou G, de Lima Morais DA, Chang L, Barrette M, et al. MetaboAnalyst 5.0: narrowing the gap between raw spectra and functional insights. Nucleic Acids Res. 2021;49:W388-W396.

25. Twelkmeyer B, Tardif N, Rooyackers O. Omics and cachexia. Curr Opin Clin Nutr Metab Care. 2017;20:181-185.

26. Gallagher IJ, Jacobi C, Tardif N, Rooyackers O, Fearon K. Omics/systems biology and cancer cachexia. Semin Cell Dev Biol. 2016;54:92-103.

27. Cao Z, Zhao K, Jose I, Hoogenraad NJ, Osellame LD. Biomarkers for Cancer Cachexia: A Mini Review. Int J Mol Sci. 2021;22:4501.
